# Attribute Discrimination Index-Based Method to Balance Attribute Coverage for Short-Length Cognitive Diagnostic Computerized Adaptive Testing

**DOI:** 10.3389/fpsyg.2020.00224

**Published:** 2020-02-28

**Authors:** Yutong Wang, Xiaojian Sun, Weifeng Chong, Tao Xin

**Affiliations:** ^1^Collaborative Innovation Center of Assessment for Basic Education Quality, Beijing Normal University, Beijing, China; ^2^School of Mathematics and Statistics, Southwest University, Chongqing, China

**Keywords:** balance attribute coverage, cognitive diagnostic computerized adaptive testing, attribute discrimination index, equalization of attribute correct classification rate, examinee qualification rate

## Abstract

We propose a new method that balances attribute coverage for short-length cognitive diagnostic computerized adaptive testing (CD-CAT). The new method uses the attribute discrimination index (ADI-based method) instead of the number of items that measure each attribute [modified global discrimination index (MGDI)-based method] to balance the attribute coverage. Therefore, the information that each attribute provides can be captured. The purpose of the simulation study was to evaluate the performance of the new method, and the results showed the following: (a) Compared with uncontrolled attribute-balance coverage method, the new method produced a higher mastery pattern correct classification rate (PCCR) and attribute correct classification rate (ACCR) with both the posterior-weighted Kullback–Leibler (PWKL) and the modified PWKL (MPWKL) item selection method. (b) Equalization of ACCR (E-ACCR) based on the ADI-based method leads to better results, followed by the MGDI-based method. The uncontrolled method leads to the worst results regardless of item selection methods. (c) Both the ADI-based and MGDI-based methods produced acceptable examinee qualification rates, regardless of item selection methods, although they were relatively low for the uncontrolled condition.

## Introduction

Cognitive diagnostic assessment (CDA) has become popular in test theory research in recent years, which is developed to measure the cognitive skills of examinees (Leighton and Gierl, 2007; Gierl et al., 2008). Compared with classical test theory (CTT) and the most commonly used unidimensional item response theory (UIRT), which only provide overall scores to examinees, and multidimensional item response theory (MIRT), which provides both overall score and subscale scores, CDA can provide more detailed information about strengths and weaknesses of examinees for a specific content domain, so that administrators can identify whether or not examinees possess the attributes (Yao and Boughton, 2007; Lee et al., 2012). Evidence should be obtained of model fit when IRT models are used in real test data, and it is the same with CDA models (Sinharay and Haberman, 2014). Otherwise, the misfit of models may lead to a misleading conclusion.

Computerized adaptive testing (CAT) combines test theory with computer technology to improve testing efficiency, which has become a promising method in psychological and educational measurement. CAT can provide equivalent or even higher measurement accuracy of examinees' latent skills, with reductions in test length of up to 50%, compared with traditional paper-and-pencil tests (Weiss, 1982). Further, items administered in the test are matched with examinees' estimated latent trait level (Mao and Xin, 2013; Chang, 2015). Recently, to maximize the benefits of both CDA and CAT, researchers have attempted to combine CDA with CAT and named it cognitive diagnostic CAT (Xu et al., 2003; McGlohen and Chang, 2008; Cheng, 2009a; CD-CAT).

In CD-CAT, many factors can affect the reliability and validity of the test, one of which is the balance of attribute-level coverage (Cheng, 2010; Mao and Xin, 2013). Cheng (2010) pointed out that it is very important to make sure that each attribute in the test has been measured adequately or the reliability of the test will not be reduced. Furthermore, test validity will be at risk because of inadequate attribute coverage (Cheng, 2010). To balance attribute coverage in CD-CAT, Cheng (2010) developed the modified maximum global discrimination index (MMGDI) to build the item selection method. The MMGDI method is based on the global discrimination index (GDI) developed by Xu et al. (2003). The mechanism of MMGDI is to accumulate the Kullback–Leibler (KL) information between conditional distribution given estimated pattern profile and conditional distribution given each of all possible candidate pattern profiles. However, there is a problem that the GDI method eliminates the coverage at the attribute level. To overcome that shortcoming, the MMGDI method uses the maximum priority index (MPI) method to balance attribute coverage (Cheng and Chang, 2009). In the simulation study, Cheng (2010) showed that the new item selection method not only improved the attribute correct classification rate (ACCR) and the rate of attribute master pattern (AMP) but also improved the validity of the test.

The findings from Cheng (2010) indicated that the correct classification rate had increased when the number of items measuring each attribute is adequate, which implied that there is a positive correlation between the numbers of items measuring each attribute and the correct classification rate. However, Finkelman et al. (2009) pointed out that, in some situations, even if the test contained adequate numbers of items to measure each attribute, different measurement accuracy could occur across the attributes. In other words, the number of items measuring each attributes maybe not the essential factor that affects the measurement accuracy of latent skills.

Note that based on the information that each item provided, CAT can produce accurate estimates of latent skills with lesser items. We can infer that the information each item provided may be the essential factor that affects the accuracy of latent skills and affects the attribute measurement precision. Consequently, we investigated the argument whether the information that each attribute provided can be utilized as the index to balance attribute coverage.

The purpose of the current study is to explore a new method based on the information provided by each attribute, instead of the number of items used in the test to measure each attribute in CD-CAT. The major benefit of this approach is to balance the attribute coverage in a short-length test. There are several reasons for choosing a short-length test: First, CDAs can be used to design as low-stake testing, and they help teachers or administrators to understand the performance of students and thus determine what should be done to improve the students' performance (Roussos et al., 2007; Hartz and Roussos, 2008; Mao and Xin, 2013; Kaplan et al., 2015). As a consequence, cognitive diagnostic tests would be conducted more frequently than traditional tests in some areas such as interim assessment (Roussos et al., 2007; Hartz and Roussos, 2008; Mao and Xin, 2013; Kaplan et al., 2015). When CD-CAT is applied to interim assessment, the AMPs of students should be obtained with short-length tests (Zheng and Chang, 2016). Second, to the best of our knowledge, among the studies focused on short-length test, there are only two applied that CD-CAT. The first one is practiced by Wang (2013), who introduced the mutual information (MI) item selection method in CD-CAT. And the second one is practiced by Zheng and Chang (2016), who developed two high-efficiency algorithms to select items in CD-CAT. But no study appears to have considered the situation that balances attribute coverage in the test.

The remainder of the present paper is organized as follows. The *Reduced Reparameterized Unified Model* section introduces the cognitive diagnostic model (CDM) that we have used in this study. The *Item Selection Methods* section presents two chosen methods, PWKL and MPWKL information for CD-CAT. After that, we introduce two methods to balance attribute coverage: one is to balance the number of items that measures each attribute and the other one is to balance the information that each attribute provides. In a further section, we report the results of a simulation study to evaluate the performance of the novel balanced attribute coverage method.

## Reduced Reparameterized Unified Model

We used the reduced reparameterized unified model (RRUM) in the current study (Hartz, 2002), because previous studies have demonstrated that its prototype, the RUM, is very useful for formative assessment in practice (Jang, 2005; Wang et al., 2011). RRUM has gained more attention for educational assessment by researchers in recent years (Kim, 2011; Feng et al., 2013; Chiu et al., 2016). Chiu et al. (2016) also pointed out that RRUM has more flexibility than the “deterministic inputs, noisy ‘and' gate” (DINA) model proposed by Junker and Sijtsma (2001). The item response function of the RRUM can be written as

(1) $$\begin{array}{r} P\left(x_{ij}=1|\alpha_{i}\right)=\pi_{j}^{*}\overset{K}{\underset{k=1}{\prod }}{r_{jk}^{*}}^{\left(1-\alpha_{ik}\right)q_{jk}}, \end{array}$$

where α_*i*_ = (α_*i*1_, α_*i*2_, …, α_*iK*_) is the AMP of examinee *i*; η_*i*_ is the residual ability parameter of examinee *i*, which represents the latent trait account for attributes that are not included in the *Q*-matrix (McGlohen and Chang, 2008); *K* is the number of attributes. $\pi_{j}^{*}$ represents the probability that examinee *i* possesses all of the required attributes for item *j* and correctly applies them, which is formulated as $\pi_{j}^{*}=\prod_{k=1}^{K}{\pi_{jk}}^{q_{jk}}$. And $r_{jk}^{*}$ represents the ratio that examinee *i* lacks attribute *k* but correctly applies it to item *j*, which can be written as *P*(*Y*_*ijk*_ = 1|α_*ik*_ = 0), and examinee *i* possesses attribute *k* and correctly applies it to item *j*, which can be written as *P*(*Y*_*ijk*_ = 1|α_*ik*_ = 1), so $r_{jk}^{*}$ can be described as

(2) $$\begin{array}{r} r_{jk}^{*}=\frac{P\left(Y_{ijk}=1|\alpha_{ik}=0\right)}{P\left(Y_{ijk}=1|\alpha_{ik}=1\right)}, \end{array}$$

where *q*_*jk*_ is the attribute that item *j* measured, and *q*_*jk*_ = 1 presents if item *j* measures attribute *k*, otherwise *q*_*jk*_ = 0.

## Item Selection Methods

### Posterior-Weighted Kullback–Leibler Information Method

KL information assumes that all candidate AMPs, α_*c*_, share $\frac{1}{2^{K}}$ probabilities equally that belong to the true AMP for each examinee at each step of item selection. Cheng (2009a,b) commented that this assumption was unnecessary and may lead to low test efficiency. Cheng also pointed out that different candidate AMPs should have different probabilities to be the true AMP, and then he proposed a new item selection method that considered the posterior probability of examinees' responses. That modified approach was termed PWKL information:

(3) $$\begin{array}{l} PWKL_{j}\left(\hat{\alpha }\right)=\\ \overset{2^{K}}{\underset{c=1}{\sum }}\left\{\left[\overset{1}{\underset{x=0}{\sum }}\log\left(\frac{P\left(X_{j}=x|\hat{\alpha }\right)}{P\left(X_{j}=x|\alpha_{c}\right)}\right)P\left(X_{j}=x|\hat{\alpha }\right)\right]L\left(\alpha_{c}|X_{t-1}\right)\right\}, \end{array}$$

and

$$\begin{array}{l} L\left(\alpha_{c}|X_{t-1}\right)\propto \left(\overset{t-1}{\underset{j=1}{\prod }}P{\left(x_{j}=1|\alpha_{c}\right)}^{x_{j}}{\left[1-P\left(x_{j}=1|\alpha_{c}\right)\right]}^{1-x_{j}}\right)p\left(\alpha_{c}\;\right), \end{array}$$

where *L*(α_*c*_|*X*_*t*−1_) is the likelihood function, *X*_*t*−1_ is response vector of *t* − 1 items, and *p*(α_*c*_) is the prior distribution of α_*c*_. The item *t* will be selected for a specific examinee with maximum PWKL information. Simulation studies have shown that PWKL information outperformed KL information and Shannon entropy (SHE) algorithms in most aspects (Cheng, 2009a,b; Wang, 2013).

### Modified Posterior-Weighted Kullback–Leibler Information Method

The MPWKL method modifies the PWKL method to lead to a more reasonable result, especially in short-length test (Kaplan et al., 2015). The PWKL method uses point estimate, whereas the MPWKL method uses the entire posterior distribution. Thus, more information can be gained from the MPWKL than the PWKL method. The MPWKL information method is shown as follows:

(4) $$\begin{array}{l} MPWKL_{ij}=\sum_{d=1}^{2^{k}}\{\sum_{c=1}^{2^{k}}[\sum_{x=0}^{1}\log\left(\frac{P\left(X_{ij}=x|\alpha_{d}\right)}{P\left(X_{ij}=x|\alpha_{c}\right)}\right)\\ P\left(X_{ij}=x|\alpha_{d}\right)\pi \left(\alpha_{c}|X_{n-1}\right)]\pi (\alpha_{c}|X_{n-1})\}. \end{array}$$

## Methods For Balancing Attribute Coverage

### Balance Attribute Coverage Based on Number of Items That Measure Each Attribute

Cheng and Chang (2009) introduced the MPI method to select items to meet the constraints in IRT-based CAT. Later, Cheng (2010) extended the MPI method to CD-CAT for balancing attribute coverage. The definition of the attribute-balance index (ABI) is

(5) $$\begin{array}{r} ABI_{j}={\overset{K}{\underset{k=1}{\prod }}\left(\frac{B_{k}-b_{k}}{B_{k}}\right)}^{q_{jk}}, \end{array}$$

where *B*_*k*_ is the lower bound of the number of items required to measure attribute *k, b*_*k*_ is the number of items measuring attribute *k* that has already been selected, and *q*_*jk*_ is the element of *Q*-matrix. The value of ABI is non-negative. By combining ABI and PWKL information methods, the modified global discrimination index (MGDI) is formulated as

(6) $$\begin{array}{l} MGDI_{j}=ABI_{j}*PWKL\left(\hat{\alpha }\right)=\overset{K}{\underset{k=1}{\prod }}\left(\frac{B_{k}-b_{k}}{B_{k}}\right)*PWKL_{j}\left(\hat{\alpha }\right) \end{array}$$

An item with maximum MGDI will be administered as the next item for a specific examinee. Cheng (2010) named it maximum MGDI (MMGDI) item selection method. It is worth noting that the MMGDI method will be used to select the next item if ABI is larger than 0; otherwise, the PWKL information method will be used. When *q*_*jk*_ = 0, which means item *j* does not measure attribute *k*, then ${\left[\frac{\left(B_{k}-b_{k}\right)}{B_{k}}\right]}^{q_{jk}}=1$, which does not affect MGDI_*j*_.

### Balance Attribute Coverage Based on Attribute Discrimination Index

As mentioned in the *Introduction*, in some situations, even though adequate items are used to measure each attribute, the estimated accuracy may differ across attributes (Finkelman et al., 2009). The number of items measuring each attribute may be the necessary condition to improve the AMPs' accuracy. However, the information that each attribute provides may also be an essential factor to increase the test accuracy. Therefore, not only measuring each attribute with the number of items but also information that each attribute provides can be used to balance attribute coverage.

Henson et al. (2008) developed the attribute discrimination index (ADI) to compute the information each attribute provided. Then Finkelman et al. (2010) developed a binary programming method based on ADI to assemble tests automatically for CDM. ADI aims to compute the expected KL information between any two AMPs, with all the attributes holding constant except the target attribute, within the ideal response pattern (IRP; Tatsuoka, 1995). Considering that the test that measures *K* attributes will produce 2^*K*^(2^*K*^ − 1) possible comparisons regardless of hierarchy among attributes, a (2^*K*^ × 2^*K*^) matrix **D**_*j*_ will be used to contain all these values. **D**_*j*_ can be written as follows:

(7) $$\begin{array}{l} D_{juv}=E_{\alpha_{u}}\left[\log\left(\frac{P_{\alpha_{u}}\left(x_{j}\right)}{P_{\alpha_{v}}\left(x_{j}\right)}\right)\right]=\\ P_{\alpha_{u}}\left(1\right)\log\left(\frac{P_{\alpha_{u}}\left(1\right)}{P_{\alpha_{v}}\left(1\right)}\right)+P_{\alpha_{u}}\left(0\right)\log\left(\frac{P_{\alpha_{u}}\left(0\right)}{P_{\alpha_{v}}\left(0\right)}\right), \end{array}$$

where *P*_α_*u*__(*x*_*j*_) and *P*_α_*v*__(*x*_*j*_) are response probabilities of item *j* given AMPs α_*u*_ and α_*v*_, respectively. *D*_*juv*_ represents the degree to which a master (non-master) differed from non-master (master) for the target attribute (Henson et al., 2008).

There are 2^(K−1)^ comparisons of AMPs that differ only for the target attribute *k*. Note that the KL information between two AMPs is not symmetric. Therefore, two ADIs can be calculated for item *j*: one is the power that discriminates the master from non-master for the target attribute and the other one discriminates the non-master from master. The formulations of these two ADIs are

(8) $$\begin{array}{r} ADI_{jk1}=\underset{\alpha_{u},\alpha_{v}\in \;\Omega_{1}}{\sum }\omega_{k1}D_{juv}, \end{array}$$

(9) $$\begin{array}{r} ADI_{jk0}=\underset{\alpha_{u},\alpha_{v}\in \;\Omega_{0}}{\sum }\omega_{k0}D_{juv}, \end{array}$$

where ω_*k*1_ = *p*(α_*u*_|α_*k*_ = 1), Ω_*k*1_ ≡ {α_*uk*_ = 1 *and α*_*vk*_ = 0 *and α*_*um*_ = α_*vn*_ ∀ *m* ≠ *n*}, and ω_*k*0_ = *p*(α_*u*_|α_*k*_ = 0), Ω_*k*0_ ≡ {α_*uk*_ = 0 *and α*_*vk*_ = 1 *and α*_*um*_ = α_*vn*_ ∀ *m* ≠ *n*}. In general, ω_*kg*_ is the weight of *D*_*juv*_. Two situations need to be considered: First, there is no idea about the prior information of examinees population; then all AMPs are equally likely, which means $\omega_{kg}=\frac{1}{2^{\left(K-1\right)}}$; second, the situation in which each AMP has different prior information and the estimates of the joint probabilities of the AMPs will be used as the weight of *D*_*juv*_ (Henson et al., 2008). Henson et al. (2008) defined the ADIs under the first situation as ADI_(A)_ and the second as ADI_(B)_. Noting that ADI_(A)_ is related to items and unrelated to the knowledge states of examinees, therefore, this index can be used to represent the degree that the attribute is being measured by items. As a consequence, the ADI_(A)_-based ABI (ADI_A_-ABI) can be defined as

(10) $$\begin{array}{r} ADI_{\left(A\right)}-ABI_{j}={\overset{K}{\underset{k=1}{\prod }}\left(\frac{ADI_{\left(A\right)k}-adi_{\left(A\right)k}}{ADI_{\left(A\right)k}}\right)}^{q_{jk}}, \end{array}$$

where *ADI*_(*A*)*k*_ is the lower bound ADI of attribute *k* and the value of *ADI*_(*A*)*k*_ is the average of *ADI*_(*A*)*k*1_ and *ADI*_(*A*)*k*0_ (Finkelman et al., 2010); *adi*_(*A*)*k*_ represents ADI of attribute *k* that has already been selected.

The difference between the number of items measuring each attribute-based (MGDI-based) ABI and ADI_(A)_-based ABI is that *B*_*k*_ and *b*_*k*_ are both positive integers and ABIs are nonnegative, whereas *ADI*_(*A*)*k*_ and *adi*_(*A*)*k*_ include any values that larger than 0. ADI_(A)_-ABI outcomes can produce negative values in some situations, which are undesirable. Hence, we constrain negative values to 0 when *ADI*_(*A*)_ − *ABI*_*j*_ < 0. By combining ADI_(A)_-ABI_*j*_ with PWKL or MPWKL information, the ADI-based item selection method can be written as

(11) $$\begin{array}{r} I_{j}\left(\hat{\alpha }\right)*\left[ADI_{\left(A\right)}-ABI\right]=I_{j}\left(\hat{\alpha }\right)*{\overset{K}{\underset{k=1}{\prod }}\left(\frac{ADI_{\left(A\right)k}-adi_{\left(A\right)k}}{ADI_{\left(A\right)k}}\right)}^{q_{jk}}, \end{array}$$

where $I\left(\hat{\alpha }\right)$ represents PWKL information or MPWKL information. If ADI_(A)_ − ABI > 0, the next item will be selected by Equation (10); otherwise, PWKL or MPWKL information method will be used to select the next item.

## Simulation Study

### Manipulated Factors

We conducted a simulation study to investigate the performance of the ADI-based method under different conditions. We manipulated four independent factors in the study.

#### Item Pool

In this study, we had designed three item pools, which all contained 775 items and measured five attributes in total. Item pools were constructed based on the study of Huebner et al. (2018) and Wang et al. (2011). In item pool 1, item parameters $\pi_{j}^{*}$ and $r_{jk}^{*}$ were generated from uniform distributions *U*(0.75, 0.95) and *U*(0.15, 0.50), respectively. Considering that $r_{jk}^{*}$ was relatively large, hence, we labeled item pool 1 as the low discrimination (LD) item pool. In item pool 2, high discrimination (HD) item pool, item parameters $\pi_{j}^{*}$ and $r_{jk}^{*}$ were generated from uniform distributions *U*(0.75, 0.95) and *U*(0.05, 0.40), respectively. In item pool 3, hybrid discrimination (HyD) item pool, item parameter $\pi_{j}^{*}$ was also generated from uniform distributions *U*(0.75, 0.95), but $r_{jk}^{*}$s were generated from uniform distributions *U*(0.05, 0.50) contained in both low and high discriminations. Tables 1, 2 present the descriptive statistics of LD, HD, and HyD item pools.

**Table 1:** Descriptive statistics of item parameters of LD item pool, HD item pool, and HyD item pool.

		π*	${\text{r}}_{1}^{*}$	${\text{r}}_{2}^{*}$	${\text{r}}_{3}^{*}$	${\text{r}}_{4}^{*}$	${\text{r}}_{5}^{*}$
LD item pool	Min	0.750	0.151	0.153	0.151	0.152	0.152
	Max	0.950	0.499	0.496	0.500	0.500	0.499
	Mean	0.848	0.327	0.326	0.328	0.335	0.329
	SD	0.058	0.100	0.101	0.100	0.099	0.107
HD item pool	Min	0.750	0.053	0.051	0.050	0.051	0.050
	Max	0.949	0.400	0.399	0.400	0.400	0.400
	Mean	0.850	0.217	0.230	0.233	0.227	0.225
	SD	0.056	0.100	0.103	0.102	0.097	0.104
HyD item pool	Min	0.750	0.052	0.051	0.051	0.052	0.052
	Max	0.950	0.495	0.500	0.499	0.498	0.498
	Mean	0.854	0.266	0.270	0.269	0.278	0.282
	SD	0.059	0.125	0.125	0.131	0.124	0.129

*LD item pool, low discrimination item pool; HD item pool, high discrimination item pool; HyD item pool, hybrid discrimination item pool*.

**Table 2:** Descriptive statistics of attribute discrimination index for each attribute of LD item pool, HD item pool, and HyD item pool.

		**A_**1**_**	**A_**2**_**	**A_**3**_**	**A_**4**_**	**A_**5**_**
LD item pool	Number of items	341	341	341	341	341
	Sum of ADI*_*k*_*	136.959	139.476	139.460	136.731	139.873
	Mean of ADI*_*k*_*	0.402	0.409	0.409	0.401	0.410
HD item pool	Number of items	400	400	400	400	400
	Sum of ADI*_*k*_*	179.699	166.479	169.338	171.138	174.643
	Mean of ADI*_*k*_*	0.449	0.416	0.423	0.428	0.437
HyD item pool	Number of items	377	355	363	382	355
	Sum of ADI*_*k*_*	173.688	165.489	164.094	167.221	151.328
	Mean of ADI*_*k*_*	0.461	0.466	0.452	0.438	0.426

*LD item pool, low discrimination item pool; HD item pool, high discrimination item pool; HyD item pool, hybrid discrimination item pool; A_1_-A_5_, attribute 1 to attribute 5; ADI, attribute discrimination index*.

#### Examinee Populations

We generated three examinee populations, each one containing 3,200 examinees. The first population (denote as *Unif* ) assumed that the AMP of each examinee, **α**, was generated from a uniform distribution of 32 possible pattern profiles with probability 1/32. Thus, each AMP had 100 examinees; meanwhile, each examinee had a 0.5 chance to master each attribute. Considering that correlations among attributes are common in practice, we used a multivariate normal distribution to describe the relationship among attributes for the second and third populations (denote as *Norm*) (de la Torre and Douglas, 2004; Cheng, 2009b; Kunina-Habenicht et al., 2012; Liu et al., 2016). The mastery probabilities for the five attributes were defined as 0.45, 0.50, 0.55, 0.60, and 0.65, respectively, in both populations. The correlations among attributes were set at 0.5 (low correlation) for the second population and 0.8 (high correlation) for the third population. Table 3 represents the frequencies of examinees who possess each possible number of attributes.

**Table 3:** Frequencies of examinees exhibiting each possible number of attributes in each population.

Number of attributes		**0**	**1**	**2**	**3**	**4**	**5**
Number of examinees	Unif	100	500	1,000	1,000	500	100
	Norm-0.5	166	250	378	489	650	1,267
	Norm-0.8	382	225	222	279	418	1,674

We obtained nine subgroups by crossing item pools and examinee populations. These combinations were as follows: LD item pool with the uniform distributed population (*LD-unif*); LD item pool with the normal distributed population and 0.5 attribute correlation (*LD-norm-0.5*); LD item pool with the normal distributed population and 0.8 attribute correlation (*LD-norm-0.8*); HD item pool with the uniform distributed population (*HD-unif*); HD item pool with the normal distributed population and 0.5 attribute correlation (*HD-norm-0.5*); HD item pool with the normal distributed population and 0.8 attribute correlation (*HD-norm-0.8*); HyD item pool with the uniform distributed population (*HyD-unif*); and HyD item pool with the normal distributed population and 0.5 attribute correlation (*HyD-norm-0.5*); and HyD item pool with the normal distributed population and 0.8 attribute correlation (*HyD-norm-0.8*).

#### Constraints of Attribute-Balance Coverage

We considered three levels of constraint: Level 1 did not constrain the coverage of attribute balance, whereas level 2 and level 3 added a constraint to it. Level 2 used the method developed by Cheng (2010), who balanced attribute coverage via the number of items measuring each attribute. In Cheng's simulation study, he set the lower bound of item number that measures each attribute at 4 (*B*_*k*_ = 4) for a 30-item test; in the current study, we set the lower bound at 2 (*B*_*k*_ = 2) for a 10-item test. Level 3 used the method proposed in the current study that balance attribute coverage via the information that each attribute provided (ADI), with 1 as the lower bound of information (*ADI*_(*A*)*k*_ = 1). The reason that setting ADI_(A)k_ = 1 was that as can be seen from Table 2, 1 was the lower bound of information for each attribute that can provide approximately two items that measure each attribute, which means level 3 and level 2 had the same constraints.

#### Item Selection Methods

Cheng (2010) used KL information method to select items successively, whereas many studies have demonstrated that PWKL information method performed better than KL information method in terms of pattern and ACCR (Cheng, 2009a,b; Mao and Xin, 2013; Wang, 2013; Hsu and Wang, 2015; Zheng and Chang, 2016). And the MPWKL information method may perform even better than PWKL (Kaplan et al., 2015). Thus, we adopted both the PWKL and MPWKL information methods in the current study.

We generated a total of 54 conditions study (3 item pools × 3 examinee populations × 3 constraints of attribute-balance coverage × 2 item selection methods). We fixed the number of items in the test to 10 in all conditions. The first item was selected randomly from the item pool, with a maximum a posteriori (MAP) method used to estimate the examinee's AMP, and the prior information of AMP assumed to follow a uniform distribution. The study procedures were implemented by R software.

### Evaluation Criteria

We evaluated results against five criteria: mastery pattern correct classification rate (PCCR), ACCR, equalization of ACCR (E-ACCR), item exposure index, and examinee qualification rate. E-ACCR is the ratio between the standard deviation of ACCR and the mean of ACCR, which represents the stability of ACCR. Examinee qualification rate means the proportion of examinees who satisfy the prescribed constraints (e.g., a minimum of two items that measure each attribute under the MGDI-based method in this study), which ranged from 0 to 1. The computation of PCCR and ACCR is as follows:

$$\begin{array}{l} PCCR=\frac{\sum_{i=1}^{N}I\left(\alpha_{i}={\hat{\alpha }}_{i}\;\right)}{N},\\ ACCR_{k}=\frac{\sum_{i=1}^{N}I\left(\alpha_{ik}={\hat{\alpha }}_{ik}\;\right)}{N}, \end{array}$$

where *N* is the number of examinees and *I*(…) is an indicator function. And item exposure index can be expressed as

$$\begin{array}{l} \chi^{2}=\overset{N}{\underset{j=1}{\sum }}\frac{{\left(\exp_{j}-\;\frac{J}{N}\right)}^{2}}{\frac{J}{N}},\\ \exp_{j}=\;\frac{N_{j}^{administered}}{N}, \end{array}$$

where *J* is the number of items, $N_{j}^{administered}$ is the number of items administered to examinees.

## Results

Table 4 lists the estimates of PCCR for each condition. The data summarized in the table make several meaningful points. First, the MPWKL information method performs similarly or even better than the PWKL information method for both LD and HD item pools, regardless of the methods that constrain attribute coverage and distribution of the population. Second, compared with uncontrolled conditions, both the PWKL and MPWKL information methods lead to better PCCR outcomes when attribute coverage was controlled, and there are only minor differences between the MGDI-based and ADI-based methods. Third, the ADI-based attribute-balance method performs better than the MGDI-based method in normal distribution populations with 0.8 attribute correlation, regardless of the quality of the item pool. Fourth, the PCCRs in HyD item pools are quite complex. Both the ADI-based and MGDI-based attribute-balance methods perform better than uncontrolled conditions. However, the MPWKL information method does not always perform better than the PWKL information method in all conditions.

**Table 4:** Results of mastery pattern correct classification rate (PCCR).

	**Uncontrolled**		**MGDI based**		**ADI based**	
	**PWKL**	**MPWKL**	**PWKL**	**MPWKL**	**PWKL**	**MPWKL**
LD-unif	0.398	0.391	0.582	0.590	0.580	0.582
LD-norm-0.5	0.470	0.458	0.579	0.579	0.591	0.598
LD-norm-0.8	0.507	0.515	0.551	0.557	0.570	0.575
HD-unif	0.378	0.410	0.705	0.706	0.675	0.675
HD-norm-0.5	0.486	0.481	0.686	0.693	0.678	0.685
HD-norm-0.8	0.579	0.578	0.678	0.682	0.692	0.702
HyD-unif	0.390	0.395	0.686	0.678	0.665	0.661
HyD-norm-0.5	0.465	0.443	0.632	0.635	0.647	0.646
HyD-norm-0.8	0.530	0.530	0.633	0.638	0.659	0.642

*Uncontrolled, attribute-balance coverage not considered; MGDI-based, balance attribute coverage via MMGDI method; ADI-based, balance attribute coverage via ADI method; PWKL, posterior-weighted Kullback–Leibler information method; MPWKL, modified posterior-weighted Kullback–Leibler information method; LD-unif, low discrimination item pool, uniform distribution of examinees and ignorable correlation among attributes; LD-norm-0.5, low discrimination item pool, normal distribution of examinees and moderate correlation among attributes, correlations among attributes set at 0.5; LD-norm-0.8, low discrimination item pool, normal distribution of examinees and moderate correlation among attributes, correlations among attributes set at 0.8; HD-unif, high discrimination item pool, uniform distribution of examinees and ignorable correlation among attributes; HD-norm-0.5, high discrimination item pool, normal distribution of examinees and moderate correlation among attributes, correlations among attributes set at 0.5; HD-norm-0.8, high discrimination item pool, normal distribution of examinees and moderate correlation among attributes, correlations among attributes set at 0.8; HyD-unif, hybrid discriminating item pool, uniform distribution of examinees and ignorable correlation among attributes; HyD-norm-0.5, hybrid discriminating item pool, normal distribution of examinees and moderate correlation among attributes, correlations among attributes set at 0.5; HyD-norm-0.8, hybrid discriminating item pool, normal distribution of examinees and moderate correlation among attributes, correlations among attributes set at 0.8*.

**Figure 1:**
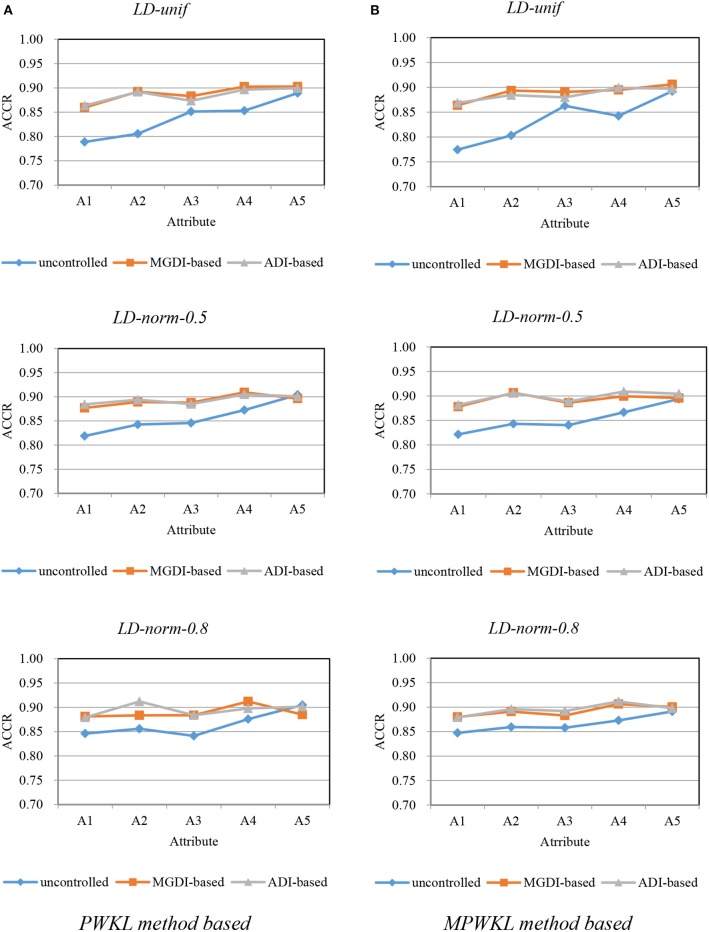
Attribute correct classification rates (ACCRs) under posterior-weighted Kullback–Leibler (PWKL) and modified PWKL (MPWKL) information methods for low discrimination (LD) item pools. **(A)** PWKL method based. **(B)** MPWKL method based.

**Figure 2:**
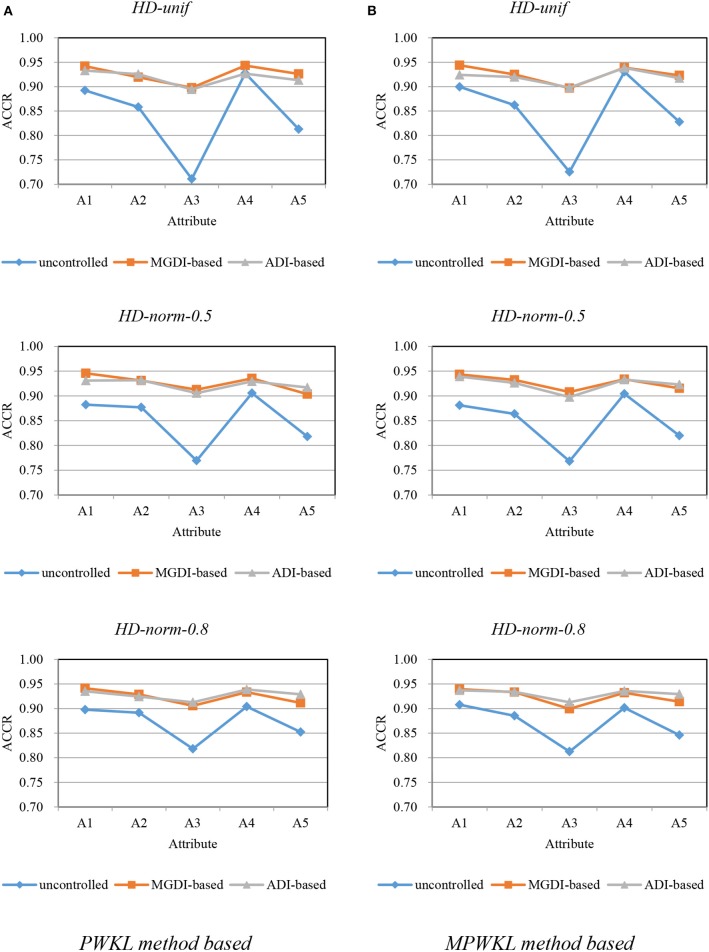
Attribute correct classification rates (ACCRs) under posterior-weighted Kullback–Leibler (PWKL) and modified PWKL (MPWKL) information methods for high discrimination (HD) item pools. **(A)** PWKL method based. **(B)** MPWKL method based.

**Figure 3:**
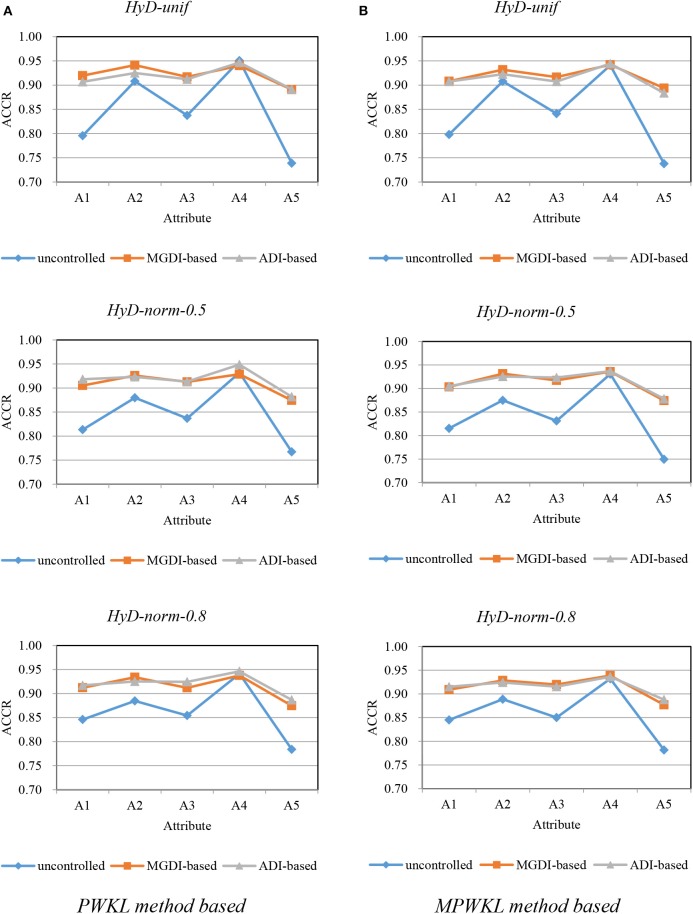
Attribute correct classification rates (ACCRs) under posterior-weighted Kullback–Leibler (PWKL) and modified PWKL (MPWKL) information methods for hybrid discrimination (HyD) item pools. **(A)** PWKL method based. **(B)** MPWKL method based.

Figures 1–3 depict the ACCR for each condition, and Table 5 represents the summary of ACCR and E-ACCR. They document the following results: First, the MPWKL information method has a similar performance or even outperforms the PWKL information method with ACCR for both LD and HD item pools with all populations under coverage controlled conditions and E-ACCR in most cases. Second, the coverage of ACCR and E-ACCR under uncontrolled conditions performs the worst, whereas they are comparable between the MGDI-based and ADI-based methods. And most of the E-ACCRs of the MGDI-based method perform slightly worse than the ADI-based method. Third, in the LD and HD item pools, when the PWKL information method was employed, the E-ACCR for uncontrolled conditions yields worse results than does the MGDI-based method; meanwhile, the ADI-based method leads to the best results. Fourth, in the HyD item pool, the ACCRs and E-ACCRs with both the ADI-based and MGDI-based attribute-balance methods outperform uncontrolled conditions; meanwhile, the ADI-based attribute-balance method performs the best under the condition of HyD-norm-0.8.

**Table 5:** Summary of ACCR and E-ACCR.

		**Uncontrolled**		**MGDI based**		**ADI based**	
		**PWKL**	**MPWKL**	**PWKL**	**MPWKL**	**PWKL**	**MPWKL**
LD-unif	M	0.838	0.835	0.888	0.890	0.885	0.886
	SD	0.040	0.047	0.018	0.016	0.016	0.013
	E-ACCR	4.773	5.629	2.027	1.798	1.808	1.467
LD-norm-0.5	M	0.857	0.853	0.892	0.893	0.894	0.898
	SD	0.033	0.028	0.012	0.011	0.009	0.012
	E-ACCR	3.851	3.283	1.345	1.232	1.007	1.336
LD-norm-0.8	M	0.865	0.866	0.889	0.892	0.895	0.895
	SD	0.026	0.017	0.013	0.011	0.013	0.012
	E-ACCR	3.006	1.963	1.462	1.233	1.453	1.341
HD-unif	M	0.840	0.849	0.926	0.926	0.919	0.920
	SD	0.084	0.079	0.019	0.018	0.015	0.015
	E-ACCR	10.000	9.305	2.052	1.944	1.632	1.630
HD-norm-0.5	M	0.850	0.847	0.926	0.927	0.923	0.924
	SD	0.056	0.054	0.017	0.015	0.011	0.016
	E-ACCR	6.588	6.375	1.836	1.618	1.192	1.732
HD-norm-0.8	M	0.873	0.871	0.924	0.924	0.928	0.930
	SD	0.037	0.040	0.015	0.017	0.010	0.010
	E-ACCR	4.238	4.592	1.623	1.840	1.078	1.075
HyD-unif	M	0.846	0.845	0.922	0.919	0.916	0.913
	SD	0.085	0.082	0.021	0.019	0.021	0.022
	E-ACCR	10.047	9.704	2.278	2.067	2.293	2.410
HyD-norm-0.5	M	0.846	0.840	0.909	0.913	0.917	0.914
	SD	0.063	0.068	0.022	0.025	0.024	0.023
	E-ACCR	7.447	8.095	2.420	2.738	2.617	2.516
HyD-norm-0.8	M	0.862	0.859	0.914	0.915	0.920	0.916
	SD	0.058	0.056	0.025	0.024	0.021	0.018
	E-ACCR	6.729	6.519	2.735	2.623	2.283	1.965

*Uncontrolled, attribute-balance coverage not considered; MGDI-based, balance attribute coverage via MMGDI method; ADI-based, balance attribute coverage via ADI method; PWKL, posterior-weighted Kullback–Leibler information method; MPWKL, modified posterior-weighted Kullback–Leibler information method; LD-unif, low discrimination item pool, uniform distribution of examinees and ignorable correlation among attributes; LD-norm-0.5, low discrimination item pool, normal distribution of examinees and moderate correlation among attributes, correlations among attributes set at 0.5; LD-norm-0.8, low discrimination item pool, normal distribution of examinees and moderate correlation among attributes, correlations among attributes set at 0.8; HD-unif, high discrimination item pool, uniform distribution of examinees and ignorable correlation among attributes; HD-norm-0.5, high discrimination item pool, normal distribution of examinees and moderate correlation among attributes, correlations among attributes set at 0.5; HD-norm-0.8, high discrimination item pool, normal distribution of examinees and moderate correlation among attributes, correlations among attributes set at 0.8; HyD-unif, hybrid discriminating item pool, uniform distribution of examinees and ignorable correlation among attributes; HyD-norm-0.5, hybrid discriminating item pool, normal distribution of examinees and moderate correlation among attributes, correlations among attributes set at 0.5; HyD-norm-0.8, hybrid discriminating item pool, normal distribution of examinees and moderate correlation among attributes, correlations among attributes set at 0.8; E-ACCR, equalization of attribute correct classification rate*.

The results of the item exposure rate and examinee qualification rate for each condition are summarized in Table 6. The following results can be drawn from the table: First, both PWKL and MPWKL information methods lead to acceptable item exposure, regardless of attribute-balance constraints, quality of item pool, and population distribution. However, the MGDI-based attribute coverage constraint gains the worst outcomes. When the ADI-based attribute coverage constraint is used, it mitigates the worst result but better than the uncontrolled attribute coverage constraint for uniform distribution populations with HD and HyD item pools. Second, compared with uncontrolled attribute coverage constraint, the examinee qualification rates of both MGDI-based and ADI-based attribute coverage constraints produce perfect results, regardless of item selection methods. In addition, MGDI-based and ADI-based attribute coverage constraints lead to consistent examinee qualification rates with both PWKL and MPWKL information methods. Moreover, an unexpected result appears that examinee qualification rates for uniform distribution populations with HD and HyD item pools are extremely low.

**Table 6:** Results of item exposure rate and examinee qualification rate for each condition.

		**Uncontrolled**		**MGDI based**		**ADI based**	
		**PWKL**	**MPWKL**	**PWKL**	**MPWKL**	**PWKL**	**MPWKL**
Item exposure rate	LD-unif	86.147	85.108	132.439	135.525	113.967	112.526
	LD-norm-0.5	78.412	80.639	117.650	116.717	99.591	98.479
	LD-norm-0.8	89.486	89.497	118.588	118.641	101.490	102.526
	HD-unif	107.043	105.655	135.684	134.428	105.052	106.532
	HD-norm-0.5	82.523	80.432	122.876	123.406	91.325	92.422
	HD-norm-0.8	97.435	98.674	130.523	129.354	96.410	96.595
	HyD-unif	108.915	106.501	140.560	137.609	105.359	106.776
	HyD-norm-0.5	77.463	77.452	127.192	127.473	91.535	91.059
	HyD-norm-0.8	86.915	86.931	128.974	128.897	92.644	93.363
Examinee qualification rate	LD-unif	0.432	0.422	1.000	1.000	1.000	1.000
	LD-norm-0.5	0.504	0.510	1.000	1.000	1.000	1.000
	LD-norm-0.8	0.580	0.574	1.000	1.000	1.000	1.000
	HD-unif	0.258	0.264	1.000	1.000	1.000	1.000
	HD-norm-0.5	0.429	0.424	1.000	1.000	1.000	1.000
	HD-norm-0.8	0.516	0.508	1.000	1.000	1.000	1.000
	HyD-unif	0.287	0.290	1.000	1.000	1.000	1.000
	HyD-norm-0.5	0.431	0.418	1.000	1.000	1.000	1.000
	HyD-norm-0.8	0.501	0.501	1.000	1.000	1.000	1.000

*Uncontrolled, attribute-balance coverage not considered; MGDI-based, balance attribute coverage via MMGDI method; ADI-based, balance attribute coverage via ADI method; PWKL, posterior-weighted Kullback–Leibler information method; MPWKL, modified posterior-weighted Kullback–Leibler information method; LD-unif, low discrimination item pool, uniform distribution of examinees and ignorable correlation among attributes; LD-norm-0.5, low discrimination item pool, normal distribution of examinees and moderate correlation among attributes, correlations among attributes set at 0.5; LD-norm-0.8, low discrimination item pool, normal distribution of examinees and moderate correlation among attributes, correlations among attributes set at 0.8; HD-unif, high discrimination item pool, uniform distribution of examinees and ignorable correlation among attributes; HD-norm-0.5, high discrimination item pool, normal distribution of examinees and moderate correlation among attributes, correlations among attributes set at 0.5; HD-norm-0.8, high discrimination item pool, normal distribution of examinees and moderate correlation among attributes, correlations among attributes set at 0.8; HyD-unif, hybrid discriminating item pool, uniform distribution of examinees and ignorable correlation among attributes; HyD-norm-0.5, hybrid discriminating item pool, normal distribution of examinees and moderate correlation among attributes, correlations among attributes set at 0.5; HyD-norm-0.8, hybrid discriminating item pool, normal distribution of examinees and moderate correlation among attributes, correlations among attributes set at 0.8*.

## Discussion and Conclusion

CD-CAT captures the advantages of both CDA and CAT, allowing the diagnosis of strengths and weaknesses of examinees with fewer items. CD-CAT can be used for low-stakes testing, so it can be adopted to provide detailed information on examinees for educators regularly (Hartz and Roussos, 2008; Mao and Xin, 2013; Kaplan et al., 2015). Thus, educators can provide remedial instruction for those examinees who need help. It is worth noting that the test length of CD-CAT should not be too long, in order to avoid increasing the burden on students. It should deviate from the original orientation by using a computer-based test to reduce students' burden and improve the efficiency of testing and learning if students do not take the test too long.

It is critical to consider the structure of short tests to assess the knowledge states of examinees comprehensively in CD-CAT. It is also important that each attribute should be measured adequately. Cheng (2010) used the number of items measuring each attribute to balance the coverage of attributes. The current study uses the information that each attribute provided to balance attribute coverage, as proposed by Henson et al. (2008). The simulation study was conducted to evaluate the performance of the new method, and the results showed that compared with the uncontrolled attribute coverage under the PWKL and MPWKL information methods, the ADI-based attribute-balance coverage method (the new method) improved both PCCR and ACCR. The reason is that when the attribute-balance coverage constraint is not controlled, some attributes may not be measured adequately; thus, the ADI is small for many examinees. Henson et al. (2008) demonstrated that the correlations are quite high between ADI and correct classification rates. Therefore, ADI can be used as the indicator of correct classification rates reasonably. Moreover, Cheng (2010) pointed out that the smallest ACCR dominated the PCCR, and he described this phenomenon as similar to Liebig's *law of the minimum*, which means the shortest stave is the most important factor that affects the capacity of a barrel with staves. In sum, considering that some ADIs are slightly smaller when attribute-balance coverage is not controlled, the ACCRs for some attributes are lower. As a consequence, the PCCRs under uncontrolled conditions are lower than those of MGDI-based and ADI-based attribute-balance coverage methods.

The present results also show that, compared with the uncontrolled method, both the ADI-based and MGDI-based attribute-balance coverage methods produce noticeable better results of PCCR and E-ACCR and slightly better ones of ACCR. Although there are no noticeable differences of E-ACCR between the ADI-based method and the MGDI-based method, the ADI-based method performs slightly better for most conditions. We infer that the ADI-based attribute-balance coverage method produces more stable ACCR than the other two methods. Besides, regardless of item selection methods, all examinees satisfied the prescribed constraints when the ADI-based and MGDI-based methods have been used, whereas the uncontrolled method failed for some examinees.

It is worth noting that when attribute-balance coverage is uncontrolled, the examinee qualification rates for HD and HyD item pools with uniform distribution populations are extremely poor under both item selection methods, for still unknown reasons. Therefore, a further study of that effect is needed.

Some future studies can be conducted to improve and enhance the application of the ADI-based attribute-balance coverage method. First, a variable-length CD-CAT can be conducted to evaluate the performance of the ADI-based method. Under variable-length CD-CAT, the measurement precision or standard error is fixed, and the number of items administered to each examinee is different. Second, there is only one RRUM model that has been used in the current study, which is a non-compensatory model. More models can be considered to verify the generalization of the ADI-based attribute-balance coverage method, especially for compensatory models. Third, the importance of each attribute to the item is assumed to be equal, but it is common that some traits are more important than others when more than one attribute is to be measured in practice (Wang et al., 2014). Thus, researchers need to take the relative importance of each attribute into account in a future study. Lastly, how to choose the lower bound of the ADI is an additional important issue. The value that has been used in the current study is a variation of the number of items measuring each attribute in the study of Cheng (2010), but how large the ADI should be to measure each attribute adequately is still unknown. Thus, studies that address the adequacy of the ADI in CD-CAT will provide some guidelines for further test administrations.

## Data Availability Statement

All datasets generated for this study are included in the article/supplementary material.

## Author Contributions

WC and TX proposed the original concept and designed the fundamental study of this study. YW and XS wrote the simulation study code and organized the article. All authors contributed to the manuscript revision.

### Conflict of Interest

The authors declare that the research was conducted in the absence of any commercial or financial relationships that could be construed as a potential conflict of interest.
